# Barriers to Adverse Drug Reaction Reporting Among Physicians, Nurses, and Pharmacists: A Scoping Review Comparing High-Income Versus Low-/Middle-Income Countries

**DOI:** 10.3390/healthcare14070930

**Published:** 2026-04-02

**Authors:** Azfar Athar Ishaqui, Rina Tripathi, Pushp Lata Rajpoot, Reham Bakhsh, Hemalatha Thanganadar, Muath Ahmed Aldomini, Salman Ashfaq Ahmad, Khalid Orayj, Narendar Kumar, Asaad Ahmed Asaad Khalil, Mohammed Ali Kaddoura, Muhammad Bilal Maqsood

**Affiliations:** 1 Department of Clinical Pharmacy, College of Pharmacy, King Khalid University, Abha 62583, Saudi Arabia; amianishaqui@kku.edu.sa (A.A.I.);; 2 Department of Pharmacy Practice, College of Clinical Pharmacy, King Faisal University, Al Hofuf 31982, Saudi Arabia; 3 Department of Public Health, College of Nursing and Health Sciences, Jazan University, Jazan 45142, Saudi Arabia; 4 Department of Health Promotion and Education, College of Public Health and Health Informatics, Umm Al Qura University, Makkah 21955, Saudi Arabia; 5 Faculty of Pharmacy, Iqra University, Karachi 75850, Pakistan; 6 Department of Pharmacy Practice, Faculty of Pharmacy, University of Sindh, Jamshoro 76080, Pakistan; 7 Quality and Performance Administration, Eastern Health Cluster, Ministry of Health, Dammam 32253, Saudi Arabia; 8 Clinical Excellence Administration, King Fahad Specialist Hospital, Dammam 32253, Saudi Arabia; 9 Quality and Performance Administration, Eastern Health Cluster, Health Holding Company, Dammam 32253, Saudi Arabia

**Keywords:** adverse drug reactions, pharmacovigilance, high-income countries, physicians, nurses, pharmacists, low-income countries

## Abstract

**Highlights:**

**What are the main findings?**

- Knowledge, awareness, and lack of formal training were the most consistently reported barriers to adverse drug reaction (ADR) reporting across all countries and professional groups.
- Fear of blame, legal consequences, and reputational risk was common in both income groups, while limited access to reporting tools/forms/IT was reported far more often in LMIC studies than in HIC studies.

**What are the implications of the main findings?**

- In low- and middle-income countries, strengthening access to reporting tools, forms, and information technology infrastructure is a prerequisite for improving ADR reporting.
- Across all settings, establishing a supportive, non-punitive reporting culture with clear feedback mechanisms is essential to sustain clinician engagement and enhance pharmacovigilance systems.

**Abstract:**

**Background and objective**: Adverse drug reactions (ADRs) cause substantial harm, and a considerable proportion may be preventable, but under-reporting persists and weakens pharmacovigilance. Spontaneous reporting depends on clinicians, yet under-reporting persists and weakens pharmacovigilance. To map barriers to adverse drug reaction reporting among physicians, a comparison of nurses and pharmacists in single-country studies was carried out between high-income countries (HICs) and low- and middle-income countries (LMICs). **Methods**: A scoping review was conducted following PRISMA-ScR guidance. PubMed and Web of Science were searched for studies published from 2016 onward. Eligible studies were single-country primary empirical studies including physicians, nurses, or pharmacists and examining ADR reporting. Only barriers that were measured or explicitly explored and reported as extractable results were included. Barriers were coded into 12 domains and summarised by income group and profession. **Results**: A total of 44 studies were included, with 18 from HICs and 26 from LMICs. Survey designs were most common. Pharmacists were the most frequently studied cadre. Knowledge and training barriers were reported in all studies in both income groups. Fear of legal or punitive concerns was reported in 13/18 (72.2%) HIC studies and 17/26 (65.4%) LMIC studies. Time and workload barriers were reported in 10/18 (55.6%) HIC studies and 11/26 (42.3%) LMIC studies. Access barriers to tools, forms, and information technology showed the clearest income group difference: these were reported in 5/18 (27.8%) HIC studies versus 16/26 (61.5%) LMIC studies. Lack of feedback or acknowledgement was reported in 8/18 (44.4%) HIC studies and 10/26 (38.5%) LMIC studies. **Conclusions**: Barriers extend beyond individual knowledge in all settings. The main income group difference was the greater prominence of reporting system access barriers in LMICs compared with workflow and time pressure barriers in HICs. Addressing fear and building a supportive non-punitive reporting culture remains a cross-cutting priority because these were common issues in both income groups and can limit reporting even when infrastructure and training exist.

## 1. Introduction

Adverse drug reactions (ADRs) remain a major cause of harm in routine care, and a substantial proportion of ADRs is considered preventable. A meta-analysis estimated that about 45% of ADRs were preventable (95% CI: 33–58%), although estimates vary by setting and methodology [1]. However, many reactions are not predictable for an individual until they occur (e.g., idiosyncratic or allergic reactions) and cannot be prevented in advance. The suspected ADRs should be documented and reported to improve medicine safety. Even when a reaction is recognised at the bedside it may never reach the national pharmacovigilance system. That gap matters because many important drug safety signals still rely on timely reports from clinicians. Recent work continues to stress the need to strengthen pharmacovigilance impact and to keep reporting systems useful for everyday practice as medicines and monitoring approaches evolve [2]. Spontaneous reporting systems sit at the centre of pharmacovigilance in many countries because they can detect rare and unexpected reactions that may not be visible in trials. Newer approaches such as electronic health record-based signal work and artificial intelligence are expanding the toolkit. Still, they do not remove the need for high-quality clinical reporting because many signals begin with a clinician deciding that an event might be important and worth reporting [2,3].

Across settings, under-reporting is repeatedly described as common and persistent. A recent systematic review update on the extent and causes of under-reporting highlights that many ADRs are not reported even when systems exist and even when professionals report positive attitudes towards reporting [4]. More recent synthesis focused on health professional awareness and practice also shows wide variation in awareness of national centres and continued low reporting experience in practice, which reinforces that under-reporting remains a live problem rather than a historical one [5]. The same bodies of evidence also show that barriers are not just individual knowledge gaps. They include practical workflow friction and uncertainty about how to report and fear-related concerns and limited feedback after reporting. These themes appear in recent reviews that examine causes and strategies for addressing under-reporting [4,5]. A separate systematic review focused on nurses also points to training needs and system-level issues as recurring explanations for low reporting, which suggests that barriers often cut across professional groups while still presenting differently in each group [6].

Physicians, nurses, and pharmacists play distinct roles in recognising and documenting suspected reactions. Physicians may be expected to diagnose and attribute causality. Nurses often observe early clinical changes and may be the first to notice a pattern during administration and monitoring. Pharmacists may identify reactions through medication review and counselling and may also support reporting pathways within hospitals and community settings. Because these roles differ, the barriers that matter most can differ too. Recent work in low-resource settings continues to describe gaps that sit at the interface of professional role and system capability, which can shape reporting behaviour in ways that simple education alone may not fix [5,7]. These gaps can be even bigger in lower-income settings where reporting systems are often limited. Many low- and middle-income countries (LMICs) are expanding access to new medicines and vaccines while pharmacovigilance capacity often develops more slowly. A recent scoping review of strategies to strengthen pharmacovigilance systems in LMICs shows that progress depends on coordinated investments in systems and workforce and in leadership and data pathways rather than relying only on individual motivation [8]. Recent scoping work focused on sub-Saharan Africa similarly reports persistent structural barriers and resource constraints alongside knowledge and process issues, which supports the view that barriers cluster differently across contexts [7].

Existing reviews provide important summaries, yet they often pool evidence across many countries and mixed populations. That can make it hard to map findings cleanly to a single income group and to isolate the barriers reported by physicians, nurses, and pharmacists when studies combine broader health worker categories. It also complicates comparisons because multi-country studies can mask country-specific reporting infrastructure and regulatory expectations. The need is therefore for evidence that is tightly linked to one country at a time and clearly tied to the professional groups who are central to spontaneous reporting. This review focuses on barriers to ADR reporting among physicians, nurses, and pharmacists within single-country studies and compares patterns between high income countries (HICs) and LMICs using World Bank income groupings. The aim is to produce an evidence map that is directly usable for policy and practice by showing which barriers are most frequently reported and how they differ by income context and by professional group.

## 2. Materials and Methods

### 2.1. Study Design and Reporting Standards

This review was conducted as a scoping review to map the empirical evidence on barriers to ADR reporting among physicians, nurses, and pharmacists across countries with different income classifications. The conduct and reporting followed established scoping review guidance and reporting standards, including the PRISMA Extension for Scoping Reviews (PRISMA-ScR) [9]. The PRISMA-ScR checklist is provided as Supplementary Material (Table S1)

### 2.2. Review Question and PCC Framework

The review question was as follows: What barriers to ADR reporting are reported by physicians, nurses, and pharmacists, and how do these barriers differ between HICs and LMICs? The PCC framework was defined a priori as follows: the population was restricted to physicians, nurses, and pharmacists. The concept was barriers to ADR reporting (i.e., reasons for under-reporting) with extractable evidence found in the results; and the context was single-country healthcare settings, enabling comparison by World Bank income group.

### 2.3. Eligibility Criteria

Studies were eligible if they met all of the following criteria: (a) primary empirical research using quantitative, qualitative, or mixed-methods designs; (b) participants included physicians and/or nurses and/or pharmacists; (c) the topic addressed ADR reporting or pharmacovigilance reporting behaviour; (d) the study was conducted in a single country (multi-site within the same country was eligible); (e) publication date fell within the last 10 years relative to the search date (restricted to 2016 onward). Restricting inclusion to 2016 onward was intended to provide a contemporary overview aligned with current pharmacovigilance practice. Reporting pathways have changed quickly in recent years with wider use of electronic reporting, so newer studies are more relevant to today’s context.

A barrier evidence requirement was applied to ensure analytic extractability: barriers had to be measured or explicitly explored in the methods and reported in Section 3 as extractable evidence (e.g., frequencies/percentages, predictors/associations, themes supported by quotations). Studies were excluded when barriers were only mentioned as a brief statement in the discussion or conclusion without supporting evidence. Additional exclusions were applied to preserve interpretability for income group comparisons: multi-country studies and cross-national surveys were excluded unless single-country results were separately reported in a way that could be extracted. Studies with mixed healthcare worker samples were excluded when results were not disaggregated and did not clearly include at least one of the three target professions, and mixed samples were included only when participants were exclusively physicians/nurses/pharmacists or when results were stratified for at least one of these professions.

### 2.4. Information Sources

Two bibliographic databases were searched: PubMed and Web of Science. No geographic restrictions were applied. PubMed was selected for biomedical coverage and Web of Science for multidisciplinary and citation indexing. CINAHL and other discipline-specific databases were not searched, which may have reduced capture of some nursing-focused studies. Searches were limited to the defined publication window (2016 onward) and applied consistently across sources.

### 2.5. Search Strategy and Limits

Search strategies combined controlled vocabulary/keywords and free-text terms covering ADR reporting and pharmacovigilance reporting behaviour, under-reporting, barriers/reasons, and the target professional groups (physicians, nurses, pharmacists). Database filters were used to apply the publication date limits. The final search set was exported from each database for record management and screening. Full database search strings for PubMed and Web of Science are provided in Supplementary Table S1.

### 2.6. Record Management, Deduplication, and Study Selection

Records from PubMed and Web of Science were merged and deduplicated prior to screening. Deduplication was conducted using standard bibliographic matching fields (including combinations of title, author, year, and DOI/identifier where available), resulting in removal of duplicate records and creation of a unique set for screening. Study selection was completed in two stages: title/abstract screening followed by full-text assessment. Two reviewers independently screened titles/abstracts and full texts. Disagreements were resolved through discussion and consensus, with a third reviewer consulted when needed. At each stage, inclusion/exclusion decisions were recorded, and reasons for exclusion at full text were documented. The screening process is summarised using a PRISMA-style flow diagram aligned with PRISMA 2020 flow-diagram conventions for documenting identification, screening, and inclusion.

### 2.7. Income Group Classification

To enable HIC vs. LMIC/other comparison, each included study was mapped to a single country and classified using the World Bank income group categories (high-income vs. all other income categories combined). When the study period was not clearly reported, the publication year was used as a proxy for income classification, and the single-country restriction ensured that each study mapped to exactly one income group [10].

### 2.8. Data Charting and Extraction

Data were charted using a structured extraction form developed for this review, consistent with scoping review practice where extraction is typically referred to as “data charting” in the PRISMA-ScR and scoping review methods literature [11]. For each included study, charted items included bibliographic and design characteristics (year, country, design), population composition (physicians, nurses, pharmacists), and barrier findings. Although facilitators were identified during charting in earlier iterations, the final synthesis presented here focused on barriers only.

### 2.9. Barrier Framework Development and Coding

Barrier findings were coded into pre-specified thematic domains that were applied consistently across studies. Domains reflected common barriers reported in ADR reporting research and were defined to support extractable and comparable synthesis: (a) knowledge/awareness/training; (b) reporting process knowledge; (c) time/workload; tools/forms/IT access; (d) fear/legal/punitive concerns; (e) lack of feedback/acknowledgement; (f) complexity/bureaucracy; (g) incentives/motivation; (h) organisational support/culture; and less frequently reported domains such as uncertainty about (i) ADR causality and (j) missing patient/clinical information. Where a study reported multiple barriers, all relevant domains where a study reported were coded.

### 2.10. Synthesis and Analysis

Synthesis was primarily descriptive, consistent with scoping review objectives to map and characterise evidence rather than estimate pooled effects. Barrier findings were summarised by income group (HIC vs. LMIC/other) and by profession. For quantitative summarisation, the presence of each barrier domain was counted across studies within each income group, and distributions were presented as study counts and percentages; where studies provided comparable quantitative reporting, median percentage signals and ranges were summarised for each barrier domain. To support interpretation across levels of influence, barriers were additionally mapped to a five-level socio-ecological structure: (a) individual (knowledge/awareness, confidence, causality uncertainty), (b) interpersonal/team (role ambiguity, diffusion of responsibility, peer norms), (c) organisational (leadership support, local reporting culture, staffing/workload environment), (d) reporting system/process (access to forms/portals/IT, unclear pathways, complexity/bureaucracy), and (e) policy/regulatory (legal/punitive climate, liability and confidentiality concerns). Coding followed how each study framed the barrier; barriers described as clinician knowledge/awareness deficits were coded as individual unless explicitly attributed to organisational or policy factors. Profession-specific visual summaries were produced by calculating, for each profession, the proportion of studies that included that profession and reported each barrier theme, enabling comparison of barrier prominence across physicians, pharmacists, and nurses.

### 2.11. Risk-of-Bias Appraisal

A formal methodological quality or risk-of-bias appraisal was not undertaken because the primary aim was to map the nature and distribution of barrier evidence rather than to estimate effects or make effectiveness claims. This approach aligns with scoping review guidance, where critical appraisal is optional depending on review purpose [11]. However, study design and key methodological limitations (e.g., self-reported survey data and cross-sectional designs) were recorded during charting and considered when interpreting the findings.

## 3. Results

### 3.1. Study Selection and Screening Results

**Figure 1:**
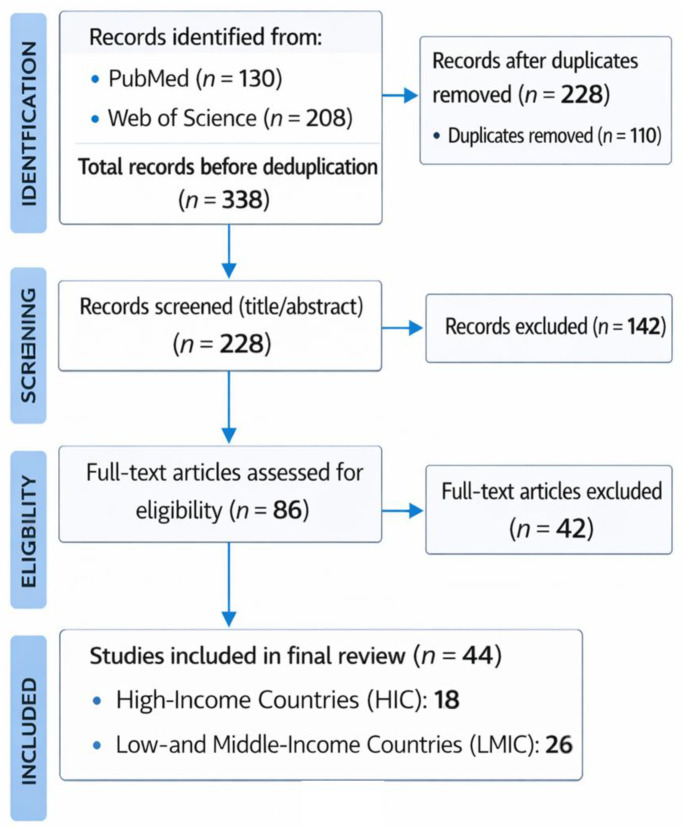
PRISMA flow diagram of study identification, screening, full-text assessment, and inclusion for the review studies.

A total of 338 records were identified across the two databases, with 130 records from PubMed and 208 records from Web of Science. After removal of 110 duplicates, 228 records were screened by title and abstract, and 142 were excluded. A total of 86 full-text articles were assessed for eligibility, and 42 were excluded at the full-text stage. In total, 44 studies were included in the final review, comprising 18 studies from HICs and 26 studies from LMICs. Figure 1 presents the PRISMA flow diagram summarising identification, screening, full-text assessment, and final inclusion.

### 3.2. Reasons for Full Text Non-Inclusion

Most of the 42 excluded full-text records were removed because they studied the wrong population. This most commonly included patient/consumer reporting studies, student populations, or healthcare cadres outside the target professions (e.g., dentists or midwives), as well as mixed healthcare worker samples where results were not disaggregated for physicians, nurses, or pharmacists. Other common reasons were not providing extractable evidence on barriers, focusing on a concept other than barriers to ADR reporting, using mixed professional samples without separate results for the target groups, or not addressing reporting behaviour. A few were excluded because they were multi-country studies or not primary empirical research.

### 3.3. Characteristics of Included Studies

A total of 42 full-text records were not included because 39 were excluded and 3 were unclear, since the participant cadre could not be confirmed from the full text. Included studies were published between 2016 and 2025. The overall median publication year was 2022. The median year was more recent in HIC studies, at 2023, while LMIC studies had a median of 2020. Most studies used quantitative cross-sectional survey designs (24 studies), followed by mixed methods designs (17 studies). Only three studies were purely qualitative, and all three were conducted in LMICs. Settings were split across hospital-only studies (22 studies) and mixed setting studies that included more than one level of care, such as hospital plus community or primary care (19 studies). Only two studies were conducted solely in community or primary care settings. Table 1 summarises the characteristics of the 44 included studies, including year, country income group, design, and study population.

Pharmacists were the most frequently studied cadre. Pharmacists appeared in 39 of 44 studies. Physicians appeared in 27 studies, and nurses appeared in 18 studies. Pharmacist-only studies accounted for 15 studies, while nurse-only studies were uncommon, accounting for two studies, and physician-only studies were rare, accounting for one study. Mixed profession designs were common overall (26 studies) and were more common in LMIC studies than in HIC studies.

### 3.4. Barriers to ADR Reporting by Domain and Income Group

Barrier findings were organised into 12 barrier domains and compared across HICs and LMICs as summarised in Table 2. The dominant barrier domain in both income groups was knowledge, awareness, and training. All HIC studies (18 of 18) and all LMIC studies (26 of 26) reported knowledge-related barriers. These barriers included limited awareness of pharmacovigilance, limited confidence in recognising ADRs, and lack of formal training on how to report. The next most frequent barrier domain in both income groups was fear and legal or punitive concerns. This domain was reported by 13/18 HIC studies and 17/26 LMIC studies. These concerns included fear of blame, fear of legal consequences, fear of harming professional reputation, and confidentiality concerns. Illustrative examples appear across multiple regions, including recent Gulf-based surveys and mixed-method studies in South Asia and Africa, with the complete list of contributing studies shown in Table 2.

Time and workload constraints were reported in 10/18 HIC studies and 11/26 LMIC studies. These barriers reflected competing clinical workload and the practical time required to complete reporting forms, particularly in high patient volume settings. Access to reporting tools and forms and information technology barriers showed one of the clearest differences by income group. This domain was reported by 5/18 HIC studies compared with 16/26 LMIC studies. In LMIC settings, this frequently reflected limited access to reporting forms, limited connectivity, lack of functional online systems, or difficulty using available tools.

Lack of feedback or acknowledgement after reporting was also common. This domain was reported by 8/18 HIC studies and 10/26 LMIC studies. These findings reflected frustration when reports were not followed by confirmation, feedback, or visible action. Unclear reporting pathways and uncertainty about who to report to and how to report were reported in 7/18 HIC studies and 8/26 LMIC studies. Complexity and bureaucracy were more common in LMIC studies, at 7/26, compared with 3/18 in HIC settings. Incentives and motivation barriers were less common overall but were more frequently reported in LMIC settings, at 4/26, compared with 1/18 in HIC settings. A small number of domains were rarely reported. Uncertainty about ADR causality appeared in only one LMIC study. Missing patient or clinical information appeared in only one HIC study. Perceived lack of seriousness or belief that the ADR was already known appeared more often in HIC studies than in LMIC studies.

### 3.5. Quantitative Signal Ranges for Common Barriers

Among studies that reported quantitative estimates, several barrier domains showed wide variation in prevalence estimates across contexts as summarised in Table 2. Knowledge and training barriers had a reported median prevalence of 40.5%, with a wide range. Time and workload barriers had a median prevalence of 55.1%. Access to tools and forms and information technology barriers had a median prevalence of 41%. Fear and punitive concern barriers had a lower median prevalence of 27.3% but still showed wide ranges, suggesting strong context dependence. Lack of feedback barriers had a lower median prevalence of 16.2%, which is consistent with feedback being salient in some systems but absent from others. These quantitative summaries should be interpreted cautiously because studies differed in instruments, question wording, sampling frames, and whether barriers were asked as multiple choice lists or measured using scaled items.

### 3.6. Barriers by Professional Group

**Figure 2:**
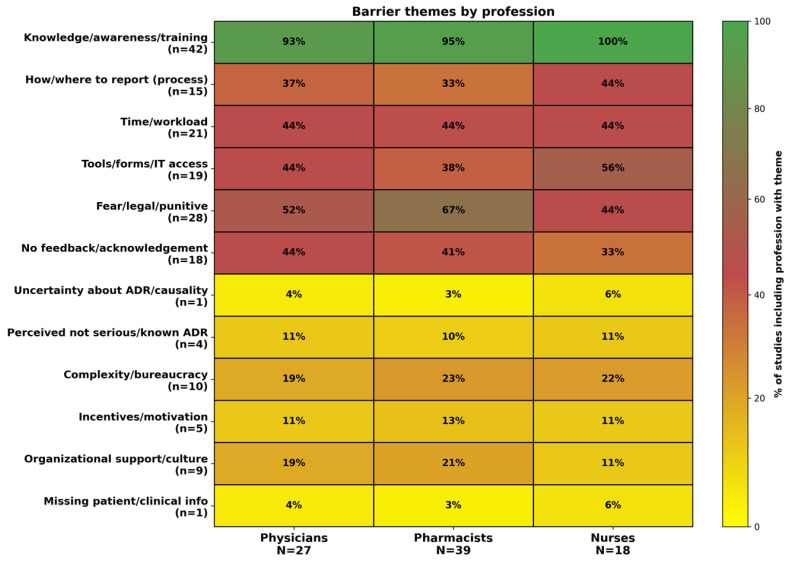
Heatmap of ADR reporting barrier themes by profession (physicians, pharmacists, nurses), showing the percentage of studies including each barrier theme for each professional group.

Figure 2 summarises barrier themes by profession using the percentage of studies that included each theme for physicians, pharmacists, and nurses. Knowledge and training barriers were present in all studies that included each professional group. Beyond knowledge barriers, there were differences in the pattern of reported barriers across professions. Among pharmacist studies, fear and punitive concerns were highly prevalent and were present in roughly seven out of ten pharmacist-inclusive studies. Time and workload and tools and information technology barriers were also frequently reported among pharmacist studies and were each present in a little under half of pharmacist-inclusive studies. Lack of feedback was also common among pharmacist-inclusive studies.

The fear-related barriers were also commonly reported among physician-focused studies. Tools and information technology barriers were reported in about half of physician-inclusive studies. Time and workload barriers and lack of feedback were each reported in just under half of physician-inclusive studies. This pattern suggests that for physicians, practical usability of the reporting system and access to tools can be as prominent as workload constraints. Among nurse studies, tools and information technology barriers were particularly prominent, appearing in over half of nurse-inclusive studies. Process barriers about how and where to report were also relatively more common in nurse-inclusive studies. Fear-related barriers and time and workload barriers were present in a little under half of nurse-inclusive studies. This pattern should be interpreted with the context in mind because nurse studies were more common in LMIC settings, which may partly explain the prominence of reporting tool access barriers.

### 3.7. Socio-Ecological Mapping of Barriers

The five socio-ecological levels were individual, interpersonal/team, organisational, reporting system/process, and policy/regulatory, and Table 3 summarises the barriers reported at each level across included studies. Individual-level barriers and reporting system- or process-level barriers were present in all included studies in both income groups. This reflects the consistent presence of knowledge and confidence barriers at the individual level and reporting pathway or tool barriers at the system level. Interpersonal- or team-level barriers were less common and appeared in 8/18 HIC studies and 8/26 LMIC studies. Organisational-level barriers were reported in 9/18 HIC and 13/26 LMIC studies, suggesting that organisational culture and institutional support issues were more frequently documented in LMIC settings. Policy- and regulatory-level barriers were reported in 12/18 HIC studies and 18/26 LMIC studies. This indicates that legal climate, regulatory expectations, and policy support were commonly raised across contexts, with a slightly higher frequency in LMIC studies.

## 4. Discussion

This review brings together evidence from 44 single-country studies, and it keeps the focus on physicians, nurses, and pharmacists only. Many included studies show that barriers are not limited to one setting or one profession because the same core issues appear in surveys and mixed-methods work from very different contexts such as Saudi Arabia, Finland, Australia, and Pakistan. The included evidence also shows that barriers are often reported together as clusters rather than isolated factors. Some studies describe knowledge gaps alongside fear and workload and system constraints in both HIC and LMIC settings. The review also adds clarity by comparing HIC and LMIC settings using studies that map to one country and one income group. This is especially helpful for system access barriers which appear frequently in LMIC contexts. Qualitative and mixed-methods papers also capture the day-to-day reality of reporting when confidence is low and workplace norms or reporting systems make it harder. Knowledge awareness and training gaps were the dominant barrier. This theme appeared in almost all included studies, and it was high in physicians, pharmacists, and nurses. This pattern suggests that under-reporting is still strongly linked to practical competence and confidence rather than a lack of interest alone. Examples were reported in HIC settings such as Finland, Australia, and Gulf countries [12,13,20,22,23,26]. Similar gaps were also reported widely in LMIC settings, including Pakistan, Nigeria, Ethiopia, and India [38,40,45,49,51,53,54].

Unclear pathways for who to report to and how to report remained a frequent barrier. This appeared in both income groups, although it was more visible in several nursing-inclusive studies, where reporting steps can be diffuse across ward routines [23,26,32,34,40]. The persistence of process confusion suggests that training alone may not be enough when reporting workflows are not embedded into everyday clinical systems. Some studies suggest that reporting responsibility is perceived as unclear, which can lead to delays or to assumptions that another team member will report [17,23]. In several settings, participants reported that they were aware of ADR reporting, but they were unsure about the correct channel or the required information, and this often discouraged reporting when time was limited [26,53].

Time and workload pressure appeared repeatedly in studies from both income groups. In HIC settings, clinicians described limited time during busy shifts and competing clinical priorities, which reduced the likelihood of completing reports, even when knowledge was adequate [22,27,28]. Similar patterns were reported in LMIC settings, where high patient volume and staffing limitations were raised as key constraints [34,36,54]. Several studies suggest that time pressure is not just a personal issue because it links to reporting burden and to how well reporting fits into routine documentation [16,51]. This suggests a gap between knowing and doing because reporting is often treated as an extra step rather than built into everyday clinical records [22,40]. A clearer implication is that in many LMIC settings, ensuring basic availability of reporting forms and functional online or mobile reporting pathways may need to come first before education- or motivation-focused strategies can produce sustained improvements in reporting behaviour.

Lack of feedback after reporting was a common theme, and it appeared across settings. Participants stated that reports were sent, but no confirmation or outcome information returned, and this reduced motivation to report again [13,37,55]. Some studies linked this barrier to trust in the system because lack of acknowledgement created a belief that reporting does not lead to action or change [34,38]. Feedback was also described as a learning mechanism because it can strengthen confidence about what to report and when to report, and it can reduce uncertainty about whether a report was appropriate [22,41]. Alongside this feedback gap, several studies also highlighted organisational support and safety culture as drivers of reporting behaviour. They described weak institutional encouragement, limited leadership emphasis, and absence of local champions who could normalise reporting as routine practice and protect staff who report the ADRs [20,24,33]. This organisational climate can shape whether feedback systems are prioritised and whether reporting feels supported or risky [20,33]. The socio-ecological mapping aligns with this interpretation because organisational- and policy-level influences were repeatedly identified, which shows that barriers extend beyond individual knowledge and include system expectations and reinforcement mechanisms [12,13].

Barrier patterns varied by professional group because nurses, pharmacists, and physicians interact with medicines and documentation systems in different ways, and this shapes what they experience as the main obstacles to ADR reporting. Nurse-inclusive studies more often highlighted practical barriers linked to reporting steps and access to tools, especially in hospital settings where escalation routes need to be clear at the point of care [17,37,40]. Pharmacist-focused studies frequently emphasised fear-related concerns, and they also described uncertainty about blame, legal exposure, and reputational risk [25,29,30,33]. Physician-inclusive studies often combined knowledge gaps with system-level barriers, such as unclear reporting pathways and limited feedback, which may reflect limited routine engagement with reporting systems outside specific environments [15,45,53]. Mixed-cadre studies reinforce that these profession patterns can coexist within the same health system, and this supports the need for profession-tailored solutions [22,26,49]. A key gap is that many studies still did not report profession-specific results when samples were mixed, which limits the precision of profession-level comparisons [49,52]. Another gap is that few studies linked the reported barriers to actual reporting frequency or facility-level reporting rates, which makes it difficult to understand which barriers have the strongest behavioural impact in each profession [13,22].

A clear difference between income groups was the prominence of tools, forms, and IT access barriers in LMIC settings, where reporting was often constrained by limited availability of forms, weak connectivity, and difficulties accessing or using reporting routes [31,34,38,49]. In HIC settings, these access barriers were present but less frequent, and the constraints more often related to workflow fit and competing time demands rather than basic availability of reporting mechanisms [15,22,23]. Workload and time pressure therefore emerged as a more visible constraint in many HIC studies, where systems and awareness were already established, but reporting still competed with clinical priorities [16,28]. Fear-related barriers remained high in both groups, which suggests that non-punitive culture and perceived legal risk are cross-cutting challenges that persist even when infrastructure is strong [12,29,35,45]. This pattern is consistent with the idea that system readiness shapes where the main friction occurs because in lower-resource contexts, barriers can begin at the first step of access while in more mature systems, barriers may shift toward workflow design and organisational reinforcement [8].

The pattern across themes suggests that single interventions are unlikely to solve under-reporting. Education may raise knowledge, but it will not remove a time burden or fear, and it may not address process confusion when reporting routes are unclear [22,40]. Simplified reporting tools can reduce bureaucracy, but they may not change a punitive culture, and they may not be used if staff do not receive feedback or do not feel protected when they report [33,53]. Feedback loops can increase engagement, but they require capacity and clear responsibility so that acknowledgement and learning messages are consistently returned to reporters [13,37]. Some included studies also point to the value of local champions and facility-level routines that normalise reporting and make it part of standard medication safety practice rather than an optional activity [20,24]. Evidence syntheses of interventions to improve reporting often point toward multi-component approaches that include education, reminders, workflow changes, and feedback [56,57,58]. The stronger emphasis on tool access in LMICs suggests that infrastructure and availability should be treated as a first step before expecting sustained behaviour change [31,34,49].

A strength of this review is the strict inclusion criteria that required extractable barrier evidence and single-country mapping to income group. This improves interpretability when comparing contexts. A limitation is that many studies used cross-sectional surveys and self-reporting, which can overemphasise knowledge gaps and under-capture organisational dynamics. Country representation was uneven (HIC studies were concentrated in Gulf/Middle East settings, with none from North America or Nordic countries in the eligible dataset), reflecting what was indexed in our searched databases and timeframe; findings should be interpreted as an evidence map rather than globally representative. Another limitation is variation in barrier measurement, which limits direct quantitative synthesis. Future work could improve comparability by using more standardised barrier instruments and by linking reported barriers to observed reporting rates. Future research should prioritise evaluation of interventions (e.g., workflow integration, reminders, feedback loops, and non-punitive governance) rather than further descriptive studies of barriers alone. More qualitative work is also needed in physician and nurse groups in some regions where evidence is sparse. Studies should also report results by profession when samples are mixed so that profession specific solutions can be designed.

## 5. Conclusions

This review shows that barriers to ADR reporting are widespread in both HIC and LMIC settings, and they extend beyond individual knowledge. Knowledge and training gaps were consistently reported across all settings, which indicates that reporting competence remains a universal need. Fear-related barriers were also common in both income groups, which suggests that concerns about blame, legal exposure, and professional reputation continue to limit reporting, even when systems are established. The main difference between income groups was the prominence of system access barriers in LMICs. Many studies from these settings described limited availability of reporting forms, weak connectivity, and difficulties accessing reporting routes, which can prevent reporting before motivation or knowledge can translate into action. In contrast, HIC studies reported tool access barriers less often and more frequently emphasised barriers linked to workflow fit, such as time pressure, competing tasks, and reporting burden within busy clinical routines. These findings suggest that improvement strategies should be prioritised differently by context. In LMIC settings, strengthening basic reporting infrastructure and ensuring accessible tools and clear reporting pathways are likely to be essential first steps. In HIC settings, where systems are typically available, the priority may shift toward integrating reporting into routine workflows and reducing time burden while maintaining supportive feedback and non-punitive culture.

## Figures and Tables

**Table 1:** Characteristics of included studies (*n* = 44) by World Bank country income group (high-income vs. low-/middle-income), with study symbol legend.

Study Symbol *	Study ID	Year	Country	Design	Main Study Population
**High-income countries (HICs) N = 18**					
☆	Aldossari et al. (2025) [12]	2025	Saudi Arabia	Cross-sectional survey	Physicians, pharmacists
✦	Alsulami (2025) [13]	2025	Saudi Arabia	Cross-sectional survey	Pharmacists
✭	Shanableh et al. (2025) [14]	2025	United Arab Emirates	Mixed methods	Pharmacists
●	Sidjimova et al. (2024) [15]	2024	Bulgaria	Survey	Physicians
☉	Khardali (2024) [16]	2024	Saudi Arabia	Mixed methods	Pharmacists
✬	Hayek et al. (2024) [17]	2024	United Arab Emirates	Mixed methods	Physicians, nurses, pharmacists
✫	Shanableh et al. (2025) [18]	2025	United Arab Emirates	Survey	Pharmacists
✪	Shareef et al. (2024) [19]	2024	United Arab Emirates	Mixed methods	Pharmacists
✧	Shanableh et al. (2023) [20]	2023	United Arab Emirates	Cross-sectional survey	Pharmacists
✯	Fossouo Tagne et al. (2022) [21]	2022	Australia	Qualitative	Pharmacists
✈	Sandberg et al. (2022) [22]	2022	Finland	Cross-sectional survey	Physicians, nurses, pharmacists
♦	Sandberg et al. (2021) [23]	2021	Finland	Cross-sectional survey	Physicians, nurses, pharmacists
⊙	Valinciute & Kubiliene (2021) [24]	2021	Lithuania	Mixed methods	Physicians, pharmacists
☯	Ali et al. (2020) [25]	2020	Saudi Arabia	Cross-sectional survey	Pharmacists
★	Li et al. (2018) [26]	2018	Australia	Survey	Physicians, pharmacists
⊗	Alsaleh et al. (2017) [27]	2017	Kuwait	Cross-sectional survey	Physicians, pharmacists
✮	Cheema et al. (2017) [28]	2017	United Kingdom	Cross-sectional survey	Pharmacists
⊘	Alharbi et al. (2016) [29]	2016	Saudi Arabia	Mixed methods	Pharmacists
**Low- and middle-income countries (LMICs) N = 26**					
■	Bahlol et al. (2025) [30]	2025	Egypt	Cross-sectional survey	Pharmacists
△	N et al. (2025) [31]	2025	India	Cross-sectional survey	Physicians, nurses, pharmacists
▽	Saleem et al. (2025) [32]	2025	Iraq	Cross-sectional survey	Physicians, nurses, pharmacists
♥	Suleiman (2025) [33]	2025	Jordan	Qualitative	Pharmacists
✉	Lirasan et al. (2025) [34]	2025	Philippines	Mixed methods	Physicians, nurses, pharmacists
♪	Issak et al. (2025) [35]	2025	Sudan	Mixed methods	Physicians, nurses, pharmacists
☒	Da Costa et al. (2025) [36]	2025	Turkey	Survey	Physicians, nurses
▼	Kabiri et al. (2024) [37]	2024	Iran	Mixed methods	Physicians, nurses
♠	Nduka et al. (2024) [38]	2024	Nigeria	Mixed methods	Pharmacists
◇	Yawson et al. (2022) [39]	2022	Ghana	Mixed methods	Physicians, nurses, pharmacists
▲	Adu-Gyamfi et al. (2022) [40]	2022	India	Mixed methods	Nurses
☑	Kitisopee et al. (2022) [41]	2022	Thailand	Qualitative	Physicians, nurses, pharmacists
⊕	Alshakka et al. (2021) [42]	2021	Yemen	Cross-sectional survey	Physicians, pharmacists
○	Andrade et al. (2020) [43]	2020	Brazil	Mixed methods	Pharmacists
✰	Rabelo Melo et al. (2020) [44]	2020	Brazil	Cross-sectional survey	Physicians, nurses, pharmacists
◆	Nadew et al. (2020) [45]	2020	Ethiopia	Mixed methods	Physicians, nurses, pharmacists
☕	Hussain et al. (2020) [46]	2020	Pakistan	Qualitative	Nurses
☘	Hussain et al. (2020) [47]	2020	Pakistan	Mixed methods	Physicians
☎	Nisa et al. (2020) [48]	2020	Pakistan	Cross-sectional survey	Physicians, pharmacists
□	Shanko and Abdela (2018) [49]	2018	Ethiopia	Cross-sectional survey	Physicians, nurses, pharmacists
♣	Udoye et al. (2018) [50]	2018	Nigeria	Cross-sectional survey	Pharmacists
☃	Hussain et al. (2018) [51]	2018	Pakistan	Mixed methods	Pharmacists
☁	Nisa et al. (2018) [52]	2018	Pakistan	Mixed methods	Physicians, pharmacists
☂	Syed et al. (2018) [53]	2018	Pakistan	Cross-sectional survey	Physicians, pharmacists
☀	Shamim et al. (2016) [54]	2016	Pakistan	Cross-sectional survey	Physicians, nurses, pharmacists
♫	Joubert and Naidoo (2016) [55]	2016	South Africa	Cross-sectional survey	Physicians, pharmacists

*: Study symbols in the first column are used to identify studies in Table 2 and Table 3.

**Table 2:** ADR reporting barrier domains in included studies comparing high-income vs. low-/middle-income countries.

High-Income Countries		ADR Reporting Barrier Domains			Low-/Middle-Income Countries	
HIC Studies n (%)	HIC Study Symbols *	Definition	Barrier Domain	(Median% Range)	LMIC Studies n (%)	LMIC Study Symbols
18/18 (100.0%)	★ ● ♦ ✈ ⊗ ⊙ ⊘ ☯ ☉ ☆ ✦ ✧ ✪ ✫ ✬ ✭ ✮ ✯	Insufficient knowledge/awareness of pharmacovigilance and ADR reporting; lack of training or educational exposure.	Knowledge/awareness/training	Median, 40.5 (range, 0.0–100.0)	26/26 (100.0%)	✰ ○ ■ □ ◆ ◇ ▲ △ ▼ ▽ ♥ ♣ ♠ ☀ ☁ ☂ ☃ ☘ ☕ ☎ ✉ ♫ ♪ ☑ ☒ ⊕
7/18 (38.9%)	♦ ✈ ⊗ ⊙ ✧ ✫ ✭	Unclear reporting pathways (who/where/how to report); unfamiliarity with reporting procedures or reporting facilitator contacts.	How/where to report (process)	Median, 26.8 (range, 20.7–68.9)	8/26 (30.8%)	◆ ▲ ▼ ▽ ♠ ✉ ♫ ☑
10/18 (55.6%)	★ ✈ ⊗ ⊙ ☉ ✧ ✪ ✬ ✭ ✮	Competing workload and time constraints that reduce capacity to complete ADR reports.	Time/workload	Median, 55.1 (range, 15.2–94.7)	11/26 (42.3%)	▲ △ ▼ ♣ ☁ ☂ ☃ ☘ ☕ ☑ ☒
5/18 (27.8%)	● ✈ ⊘ ☆ ✯	Lack of access to reporting tools (forms, online portals), poor IT/internet access, or difficulty obtaining/using reporting materials.	Tools/forms/IT access	Median, 41.0 (range, 6.3–80.0)	16/26 (61.5%)	✰ ■ □ ▲ △ ♥ ♠ ☁ ☂ ☃ ☕ ☎ ✉ ♪ ☒ ⊕
13/18 (72.2%)	★ ● ⊙ ⊘ ☯ ☉ ☆ ✧ ✪ ✫ ✬ ✭ ✮	Fear of blame, legal consequences, punishment, or reputational harm; confidentiality concerns.	Fear/legal/punitive	Median, 27.3 (range, 2.2–97.0)	17/26 (65.4%)	○ ■ ◆ △ ▽ ♥ ♠ ☀ ☁ ☂ ☃ ☘ ☕ ☎ ✉ ♪ ⊕
8/18 (44.4%)	★ ● ✈ ⊙ ☆ ✦ ✧ ✭	Lack of feedback or acknowledgement after reporting; perception that reports are not acted upon.	No feedback/acknowledgement	Median, 16.2 (range, 1.0–100.0)	10/26 (38.5%)	■ □ ▼ ♥ ♠ ☘ ✉ ♫ ♪ ☑
0/18 (0.0%)		Uncertainty about whether the reaction is drug-related; difficulty confirming causality or diagnosis.	Uncertainty about ADR/causality		1/26 (3.8%)	☑
3/18 (16.7%)	☆ ✬ ✮	Perception that ADR is mild, expected, or already well known; belief reporting is unnecessary.	Perceived not serious/known ADR	Median, 39.7 (range, 7.2–98.6)	1/26 (3.8%)	◆
3/18 (16.7%)	⊙ ✫ ✭	Reporting is perceived as complex, cumbersome, lengthy, or bureaucratic paperwork.	Complexity/bureaucracy	Median, 26.0 (range, 10.4–48.3)	7/26 (26.9%)	✰ △ ♥ ☂ ☃ ☕ ✉
1/18 (5.6%)	⊗	Low motivation or perceived lack of benefit; lack of incentives or rewards for reporting.	Incentives/motivation	Median, 60.7 (range, 26.6–72.8)	4/26 (15.4%)	△ ♣ ♠ ☑
4/18 (22.2%)	⊙ ☆ ✧ ✭	Insufficient institutional support, leadership, policies, or culture encouraging reporting.	Organisational support/culture		5/26 (19.2%)	○ ♥ ☘ ☑ ☒
1/18 (5.6%)	♦	Incomplete patient/clinical information or records needed to complete ADR reports.	Missing patient/clinical info		0/26 (0.0%)	

* Study symbols correspond to Table 1.

**Table 3:** Socio-ecological mapping of ADR reporting barriers (HIC vs. LMIC).

Study	Symbol *	Individual Level	Interpersonal/Team Level	Organisational Level	Reporting System/Process Level	Policy/Regulatory Level
**HIC Studies**						
Aldossari et al. (2025) [12]	☆	✓		✓	✓	✓
Alsulami (2025) [13]	✦	✓	✓	✓	✓	
Shanableh et al. (2025) [14]	✭	✓	✓	✓	✓	✓
Hayek et al. (2024) [17]	✬	✓	✓		✓	✓
Khardali (2024) [16]	☉	✓			✓	✓
Shanableh et al. (2025) [18]	✫	✓			✓	✓
Shareef et al. (2024) [19]	✪	✓	✓	✓	✓	✓
Sidjimova et al. (2024) [15]	●	✓			✓	✓
Shanableh et al. (2023) [20]	✧	✓	✓	✓	✓	✓
Fossouo Tagne et al. (2022) [21]	✯	✓			✓	
Sandberg et al. (2022) [22]	✈	✓		✓	✓	
Sandberg et al. (2021) [23]	♦	✓		✓	✓	
Valinciute & Kubiliene (2021) [24]	⊙	✓	✓	✓	✓	✓
Ali et al. (2020) [25]	☯	✓	✓		✓	✓
Li et al. (2018) [26]	★	✓			✓	✓
Alsaleh et al. (2017) [27]	⊗	✓	✓	✓	✓	
Cheema et al. (2017) [28]	✮	✓			✓	✓
Alharbi et al. (2016) [29]	⊘	✓			✓	
**LMIC Studies**						
Bahlol et al. (2025) [30]	■	✓	✓	✓	✓	✓
Da Costa et al. (2025) [36]	☒	✓		✓	✓	
Issak et al. (2025) [35]	♪	✓		✓	✓	✓
Lirasan et al. (2025) [34]	✉	✓		✓	✓	✓
N et al. (2025) [31]	△	✓			✓	✓
Saleem et al. (2025) [32]	▽	✓			✓	✓
Suleiman (2025) [33]	♥	✓	✓	✓	✓	✓
Kabiri et al. (2024) [37]	▼	✓		✓	✓	
Nduka et al. (2024) [38]	♠	✓		✓	✓	✓
Adu-Gyamfi et al. (2022) [40]	▲	✓	✓		✓	
Kitisopee et al. (2022) [41]	☑	✓		✓	✓	✓
Yawson et al. (2022) [39]	◇	✓	✓		✓	✓
Alshakka et al. (2021) [42]	⊕	✓			✓	✓
Andrade et al. (2020) [43]	○	✓		✓	✓	✓
Hussain et al. (2020) [47]	☘	✓	✓	✓	✓	✓
Hussain et al. (2020) [46]	☕	✓			✓	✓
Nadew et al. (2020) [45]	◆	✓	✓	✓	✓	✓
Nisa et al. (2020) [48]	☎	✓			✓	✓
Rabelo Melo et al. (2020) [44]	✰	✓			✓	
Hussain et al. (2018) [51]	☃	✓	✓	✓	✓	✓
Nisa et al. (2018) [52]	☁	✓			✓	✓
Shanko and Abdela (2018) [49]	□	✓		✓	✓	
Syed et al. (2018) [53]	☂	✓			✓	✓
Udoye et al. (2018) [50]	♣	✓	✓		✓	
Joubert and Naidoo (2016) [55]	♫	✓			✓	
Shamim et al. (2016) [54]	☀	✓			✓	

where ✓ indicates that at least one barrier in that socio-ecological level was reported in the study while blank cells indicate not reported. * Study symbols correspond to Table 1.

## Data Availability

No new data were created or analysed in this study. Data sharing is not applicable to this article.

## Supplementary Materials

The following supporting information can be downloaded at: https://www.mdpi.com/article/10.3390/healthcare14070930/s1, Table S1: Preferred Reporting Items for Systematic reviews and Meta-Analyses extension for Scoping Reviews (PRISMA-ScR) Checklist.

## Abbreviations

The following abbreviations are used in this manuscript:

ADRs	Adverse drug reactions
HICs	High-income countries
LMICs	Low- and middle-income countries
PRISMA-ScR	Preferred Reporting Items for Systematic Reviews and Meta-Analyses Extension for Scoping Reviews
PCC	Population–concept–context
DOI	Digital object identifier
